# Circulating Fibroblast Growth Factor 21 Levels Are Closely Associated with Hepatic Fat Content: A Cross-Sectional Study

**DOI:** 10.1371/journal.pone.0024895

**Published:** 2011-09-16

**Authors:** Hongmei Yan, Mingfeng Xia, Xinxia Chang, Qiong Xu, Hua Bian, Mengsu Zeng, Shengxiang Rao, Xiuzhong Yao, Yinfang Tu, Weiping Jia, Xin Gao

**Affiliations:** 1 Department of Endocrinology and Metabolism, Zhongshan Hospital, Fudan University, Shanghai, China; 2 Department of Radiology, Zhongshan Hospital, Fudan University, Shanghai, China; 3 Department of Endocrinology and Metabolism, Shanghai Clinical Center for Diabetes, Shanghai Diabetes Institute, Shanghai Jiao Tong University Affiliated Sixth People's Hospital, Shanghai, China; University of Hong Kong, China

## Abstract

**Background and Aims:**

Fibroblasts growth factor 21 (FGF21), a liver-secreted endocrine factor involved in regulating glucose and lipid metabolism, has been shown to be elevated in patients with non-alcoholic fatty liver disease (NAFLD). This study aimed to evaluate the quantitative correlation between serum FGF21 level and hepatic fat content.

**Methods:**

A total of 138 subjects (72 male and 66 female) aged from 18 to 65 years with abnormal glucose metabolism and B-ultrasonography diagnosed fatty liver were enrolled in the study. Serum FGF21 levels were determined by an in-house chemiluminescence immunoassay and hepatic fat contents were measured by proton magnetic resonance spectroscopy.

**Results:**

Serum FGF21 increased progressively with the increase of hepatic fat content, but when hepatic fat content increased to the fourth quartile, FGF21 tended to decline. Serum FGF21 concentrations were positively correlated with hepatic fat content especially in subjects with mild/moderate hepatic steatosis (*r* = 0.276, *p* = 0.009). Within the range of hepatic steatosis from the first to third quartile, FGF21 was superior to any other traditional clinical markers including ALT to reflect hepatic fat content. When the patients with severe hepatic steatosis (the fourth quartile) were included, the quantitative correlation between FGF21 and hepatic fat content was weakened.

**Conclusions:**

Serum FGF21 was a potential biomarker to reflect the hepatic fat content in patients with mild or moderate NAFLD. In severe NAFLD patients, FGF21 concentration might decrease due to liver inflammation or injury.

## Introduction

Fibroblast growth factor 21 (FGF21) belongs to a distinct “endocrine” subgroup within the FGF superfamily, consisting of FGF19, FGF21 and FGF23 [1]–[3]. Due to the lack of the conventional FGF-heparin binding domain, these FGFs can escape the body's vast deposition of heparansulphate proteoglycans and can be released into circulation and function as endocrine factors [4].

FGF21 is predominantly synthesized in liver, where it is induced by the peroxisome proliferator-activated receptors, PPARα [5]and PPARγ [6]. In addition, the expression of FGF21 is also present in pancreas, adipose, and muscle [7]–[10], FGF21 acts via FGF receptors(FGFR), though the FGFR is widely distributed in almost any tissue in the body, it is anticipated that FGF21 functions in a selective set of tissues including liver, adipose and pancreas, where β Klotho, a cofactor for FGF21 to activate FGFR, is expressed selectively [10], [11]. Physiologically, elevated FGF21 in liver can induce gluconeogenesis, fatty acid oxidation and ketogenesis in the context of prolonged fasting and starvation [12].

FGF21 has been shown to be an important protective factor against various glucose and lipid metabolic disorders in animal models [13]–[15]. For example, FGF21 activates glucose uptake in adipocytes and protectes animals from diet-induced obesity [13]. Transgenic overexpression of FGF21 improves insulin sensitivity, reduces blood glucose and triglyceride to near normal levels in both ob/ob and db/db mice [13]. Similarly, in diabetic rhesus monkeys, FGF21 significantly decreases fasting glucose, insulin, glucagon and triglycerides [14]. A recent study showed that treatment of recombinant murine FGF21 exerts beneficial effects on hepatic steatosis [15].

Several recent studies have also examined the role of FGF21 in humans, though none of these studies directly supports the metabolic regulation role of FGF21. Circulating FGF21 concentrations are increased in subjects who were either overweight or had type 2 diabetes or impaired glucose tolerance [16], [17]. Mai et al. showed that both lipid infusion and artificial hyperinsulinemia increase FGF21 levels in vivo [18]. However, another study found that the function of FGF21 is closely related to lipid metabolism instead of insulin sensitivity in humans [19]. FGF21 levels also correlate with gamma-glutamyl transferase(γ-GT) and aspartate aminotransferase(AST), indicating the close relationship between FGF21 and liver diseases [19].

Since liver is the major site for FGF21 expression and hepatic steatosis is highly correlated with impairment of glucose and lipid metabolism in humans, the relationship between hepatic steatosis and FGF21 has been investigated in several recent studies. Li et al. [20] reported that serum FGF21 levels were significantly higher in the non-alcoholic fatty liver disease (NAFLD) group compared with the controls and had a high positive correlation with intrahepatic triglyceride content(*r* = 0.662, *p*<0.001). This study, along with recent reports by Dushay et al. [21] and Yilmaz et al. [22] contributed greatly to expand our knowledge on plasma FGF21 levels in patients with NAFLD, and indicate the role of FGF21 in regulating hepatic lipid metabolism.

Although the aforementioned studies suggest that FGF21 could be a potential biomarker to screen or monitor NAFLD patients [23], the methods utilized to assess the severity of hepatic steatosis, such as B-mode ultrasound or pathological score system, were qualitative or semi-quantitative and did not reflect the quantitative association between serum FGF21 and hepatic fat content accurately. Moreover, in the study by Li et al., liver biopsies were obtained from patients undergoing resection for benign liver disease and the number of patients with precise information of hepatic fat content was rather small, which might preclude a reliable conclusion [20].

In the current study, we used ^1^H Magnetic Resonance Spectroscopy (^1^H MRS) to quantify hepatic fat content in a relatively large number of participants with impaired glucose metabolism and without known liver disease except for different degree of hepatic steatosis, and further analyzed the quantitative association between serum FGF21 level and hepatic fat content.

## Methods

### Ethics Statement

The study was approved by the human research ethics committee of Zhongshan hospital, and was conducted according to the principles of the Declaration of Helsinki. Written informed consent was obtained from all subjects.

### Subjects

The subjects were participants from a clinical intervention study named Role of Pioglitazone and Berberine in the Treatment of Non-Alcoholic Fatty Liver Disease (http://clinicaltrials.gov/, NCT00633282), which was an open, randomized, controlled clinical trial. From March 2008 to July 2010, 160 subjects (88 men and 72 women) were recruited initially from the outpatients department of endocrinology, Shanghai Zhongshan Hospital, China. All participants were diagnosed as impaired glucose regulation(including impaired fasting glucose or impaired glucose tolerance or both) or newly diagnosed diabetes and fatty liver by B ultrasonography during clinical screening tests according to the inclusion criteria of the clinical trial. No subjects took anti-diabetic medications(see exclusion criteria below). (details on the inclusion criteria of the clinical trial: http://clinicaltrials.gov/ct2/show/NCT00633282?term=NCT00633282&rank=1).

Szczepaniak and colleagues [24] had analyzed the distribution of hepatic fat content (HFC) in 2,349 participants from the Dallas Heart Study by ^1^H MRS and found 5.56% could be considered a cut-off for NAFLD. According to the study , we took HFC >5.56% as a criteria for diagnosis of NAFLD in our study too.

All subjects underwent comprehensive physical examinations, routine biochemical analyses of blood, 75g oral glucose tolerance test, hepatitis B surface antigen, hepatitis C virus antibody and ^1^H MRS. All participants completed a uniform questionnaire containing questions about the histories of present and past illnesses and medical therapy. Subjects with the following conditions were excluded from this study: (1) alcohol consumption≥140 g/week for men or 70 g/week for women; (2) acute or chronic virus hepatitis; (3)biliary obstructive diseases; (4)drug-induced liver disease; (5) total parenteral nutrition;(6) autoimmune hepatitis; (7) Wilson's disease; (8) known hyperthyroidism or hypothyroidism; (8) presence of cancer; (9) current treatment with systemic corticosteroids; (10) patients who have taken or are taking oral hypoglycemic or hypolipidemic drugs and (11) pregnancy. As the intensity of interventions in the clinical trial mentioned above was mild, patients with obvious metabolic abnormalities were excluded for the health of patients, including diabetics patients with hemoglobin A1c (HbA1c)>7.5% on initial visit ; serum triglyceride ≥5.0 mmol/L and patients with significantly impaired liver function [Alanine aminotransferase(ALT) or AST≥150 U/L]. Among the 160 subjects, the study was performed on 138 subjects (72 men and 66women) aged from 18 to 65 years old excluding 22 subjects who met the above exclusion criteria.

### Anthropometric and biochemical measurements

Body mass index (BMI) was calculated as the weight in kilograms divided by the square of the height in meters. Waist circumference was measured at the midpoint between the inferior costal margin and the superior border of the iliac crest on the midaxillary line. Waist–hip ratio (WHR) was calculated as waist circumference divided by hip circumference. Blood pressure(BP) was measured three times with 5 minute intervals each time in the seated position with a mercury sphygmomanometer in the morning,The first and fifth Korotkoff sounds were used to designate systolic(SBP) and diastolic BP(DBP), respectively. and the average of the three BPs was used as the final BP.

The biochemical indexes were measured on a Hitachi 7600 analyzer (Hitachi, Tokyo, Japan). Serum fasting glucose (FBG) and 2 hour glucosewere measured by the glucose oxidase method. Serum levels of total cholesterol (TC), triglycerides (TG), high density lipoprotein cholesterol (HDL-c), and low density lipoprotein cholesterol (LDL-c) were determined enzymatically. Apolipoprotein A, B, E (APOA, APOB, APOE) were measured by the immunoturbidimetric assay. ALT, AST,γ-GT and lactate dehydrogenase(LDH)were measured by standard enzymatic methods. HbA1c was measured by high performance liquid chromatography with HLC-723G7 automated glycohemoglobin analyzer (Tosoh, Tokyo, Japan).

### Measurement of serum FGF21

Circulating FGF21 concentrations were measured with an in-house chemiluminescence immunoassay [25] (Antibody and Immunoassay Services, University of Hong Kong). The assay was proven to be highly specific to human FGF21 and did not cross-react with other members of the FGF family (for details see Supplement S1).

### Measurement of HFC

Localized proton magnetic resonance spectroscopy (^1^H MRS) images of the liver were acquired using a 1.5-T Avanto MR system (Siemens AG, Erlangen, Germany) by an experienced radiologist. Sagittal, coronal, and axial slices through the right lobe of the liver were acquired, and an 8 cm^3^ volume of liver parenchyma was selected for further study. Spectra were collected using a Q-body coil for radiofrequency transmission and signal reception and a double-echo point-resolved spectroscopy sequence for 128 acquisitions. Areas of resonances from protons of water and methylene groups in fatty acid chains were obtained with a time-domain nonlinear fitting routine using commercial software (Syngo spectroscopy VB15, Siemens AG). HFC was calculated by dividing the integral of the methylene groups in fatty acid chains of the hepatic triglycerides by the sum of methylene groups and water [26].

### Statistical analysis

Statistical analyses were performed with SPSS software version 13.0 (SPSS, Inc. Chicago, IL). Normally distributed data were expressed as means ± SD. Data that were not normally distributed, as determined using Kolmogorox–Smirnov test, were logarithmically transformed before analysis and expressed as median with interquartile range. One-way ANOVA was used for comparisons among groups, and multiple testing was corrected using LSD method (Equal Variances Assumed) or Games-Howell method (Equal Varance not assumed). Pearson's correlations and multiple stepwise regression analysis were used to examine the association of HFC, serum FGF21, and other parameters. In all statistical tests, *p* values <0.05 were considered significant.

## Results

Among 138 subjects, 76 had impaired glucose regulation(FBG ≥5.6 mmol/L and/or a two hour glucose value ≥7.8 mmol/L) and 62 had newly diagnosed diabetes(FBG≥7.0 mmol/L and/or a two hour glucose value≥11.1 mmol/L).

Hepatic fat contents (HFCs) of all the study subjects determined by ^1^H MRS were distributed normally from 2.47% to 81.95% with a mean and standardized deviation of 32.30% and 15.95%, respectively. Using HFC >5.56% as a criteria for diagnosis of NAFLD [24], 136 subjects of the study was NAFLD.

### The general characteristics of the subjects (Table 1)

**Table 1:** The general characteristics of the study subjects.

	Hepatic fat Content (%)				
Characteristics	Q 1<22.03%(n = 41)	Q 222.04%–29.75%(n = 31)	Q 329.76%–44.57%(n = 37)	Q 4≥44.58%(n = 29)	*P* value
male/female	24/17	21/10	16/21	11/18	0.020
Age (years)	51.61±8.63	49.81±9.19	48.04±9.81	53.11±10.60	0.228
BMI (kg/m^2^)	26.26±2.49	27.77±4.06	28.27±4.18*	27.67±3.72	0.205
Waist (cm)	92.10±6.47	94.30±12.04	95.59±9.71	93.69±8.55	0.569
WHR	0.93±0.05	0.94±0.08	0.94±0.06	0.94±0.06	0.995
SBP (mmHg)	122.46±15.64	129.52±12.53	122.30±12.10	126.62±16.04	0.169
DBP (mmHg)	77.73±6.71	82.98±11.76*	79.28±7.54	79.51±11.10	0.213
FBG (mmol/L)	6.44±1.17	6.44±1.13	6.02±0.74	6.28±0.80	0.319
2hBG (mmol/L)	11.56±3.36	10.27±3.15	10.19±2.79	11.55±3.01	0.163
HbA1c(%)	6.27±0.79	6.43±0.71	6.28±0.62	6.44±0.65	0.707
TC(mmol/L)	4.96±0.79	5.14±0.85	5.47±0.74*	5.40±0.98	0.091
TG (mmol/L)	1.97±1.07	2.03±0.78	2.16±0.95	2.32±0.88	0.260
HDL-c (mmol/L)	1.13±0.24	1.12±0.23	1.20±0.28	1.18±0.23	0.576
LDL-c (mmol/L)	3.14±0.97	3.21±0.91	3.34±0.77	3.17±0.89	0.839
APOA (g/L)	1.20±0.23	1.20±0.21	1.29±0.26	1.29±0.22	0.166
APOB (g/L)	0.94±0.22	0.96±0.16	1.03±0.16*	1.05±0.24*	0.071
APOE (mg/L)	44.91±11.42	47.97±12.50	49.33±11.89	54.13±13.74*	0.063
ALT (U/L)	27.52±15.20	37.04±17.45*	43.28±22.16*	61.11±37.27* #	0.000
AST (U/L)	22.32±6.94	25.25±9.66	29.21±10.54*	37.07±15.50* # &	0.000
γ-GT (U/L)	41.11±36.10	50.86±36.94	38.31±21.82	59.96±42.99* &	0.057
LDH (U/L)	181.27±26.00	212.86±84.70	193.29±31.28	205.96±34.91*	0.066

*: Compared with group Q1 p<0.05;

# : Compared with group Q2 p<0.05;

& : Compared with group Q3 p<0.05.

BMI: Body mass index;WHR: waist-to-hip ratio; SBP: systolic blood pressure; DBP: diasystolic blood pressure; FBG: fasting blood glucose; 2hBG: 2 h postload blood glucose. TC: total cholesterol; TG: triglyceride; HDL-c: high density lipoprotein cholesterol; LDL-c: low density lipoprotein cholesterol; APOA: apolipoprotein A; APOB: apolipoprotein B; APOE: apolipoprotein E; ALT: Alanine aminotransferase; AST: Aspartate aminotransferase; γ-GT: γ-glutamyl transpeptidase; LDH: lactate dehydrogenase.

By dividing the distribution of HFC into quartile, we found that there were more male subjects than female subjects in groups with lower hepatic fat content (Q1 and Q2), but in groups with higher hepatic fat content (Q3 and Q4) there were more female subjects.

The four groups did not differ in most of metabolic parameters, except that ALT and AST were elevated gradually with the increase of HFC (both *p*<0.001). ALT was 27.52±15.20 U/L, 37.04±17.45 U/L, 43.28±22.16 U/L, 61.11±37.27 U/L and AST was 22.32±6.94 U/L, 25.25±9.66 U/L, 29.21±10.54 U/L, 37.07±15.50 U/L when HFC was in Q1,Q2,Q3,Q4, respectively. and γ-GT showed a tendency to increase when HFC increased gradually (*p* = 0.057), with highest value up to 59.96 U/L in the fourth quartile, but a obvious drop to 38.31 U/L in the third quartile.

### HFC and FGF21

With the increase of HFC, serum FGF21 also increased progressively in patients with HFC no more than the fourth quartile. The FGF21 concentrations were 194.12±126.96 pg/ml, 219.65±141.74 pg/ml and 326.44±149.47 pg/ml when HFC was in Q1, Q2, Q3, respectively. Interestingly, once HFC further increased to the fourth quartile, FGF21 tended to decline to 258.75±124.69 pg/ml. (compared with the third quartile, *p* = 0.059) (Figure S1A).

In light of the fact that FGF21 increased progressively when HFC was increased from the first quartile to the third quartile, but decreased in the fourth quartile, we analyzed the association between serum FGF21 concentration and HFC in subjects within the first three quartiles of HFC and all subjects, respectively (Figure S2). When HFC wasin Q1 to Q3, there was a significant positive association between FGF21 and HFC (*r* = 0.276, *p* = 0.009); However, the significant association between HFC and FGF21 no longer existed when HFC was in Q4 (*r* = −0.087, *p* = 0.671).

Also, we analyzed the association between HFC and other parameters in subjects within the first three quartiles of HFC and all subjects, respectively. In univariate correlation analyses, HFC in Q1–Q4 positively associated with AST (*r* = 0.487, *p*<0.001), ALT (*r* = 0.436, *p*<0.001), LDH (*r* = 0.325, *p* = 0.001), TG (*r* = 0.296, *p*<0.001), APOE(*r* = 0.252, *p* = 0.011);γ-GT(*r* = 0.238, *p* = 0.005), TC (*r* = 0.211, *p* = 0.014), APOA (*r* = 0.200, *p* = 0.028); APOB (*r* = 0.199, *p* = 0.028), and sex (*r* = 0.172, *p* = 0.043). After adjustment for sex, age and BMI, HFC in Q1–Q4 still positively associated with AST(*r* = 0.461, *p*<0.001)) ALT (*r* = 0.443, *p*<0.001), APOB (*r* = 0.277, *p* = 0.029), and TC (*r* = 0.272, *p* = 0.033). Similarly, HFC in Q1–Q3 positively associated with ALT (*r* = 0.378, *p*<0.001), AST (*r* = 0.373, *p*<0.001), TG (*r* = 0.325, *p*<0.001), LDH (*r* = 0.244, *p* = 0.026), APOB (*r* = 0.241, *p* = 0.019), TC (*r* = 0.214, *p* = 0.026), and age (*r* = −0.206, *p* = 0.033). After adjustment for sex, age and BMI, HFC in Q1–Q3 still positively associated with ALT(*r* = 0.402, *p* = 0.008) APOB (*r* = 0.350, *p* = 0.021) AST (*r* = 0.339, *p* = 0.026). Differently, HFC in Q4 negatively associated only with WHR (*r* = −0.419, *p* = 0.024) and the association was insignificant after adjustment for sex, age and BMI (Table 2).

**Table 2:** Correlations of HFC with serum FGF21 and other parameters.

Variables	HFC(Q1–4)				HFC(Q1–3)				HFC(Q4)			
	*r*	*p*	*r* *	*p* *	*r*	*p*	*r* *	*p* *	*r*	*p*	*r* *	*p* *
Sex(1 = M;2 = F)	0.172	0.043	−	−	0.087	0.369	−	−	0.119	0.539	−	−
Age	−0.009	0.916	−	−	−0.206	0.033	−	−	0.050	0.798	−	−
BMI	0.110	0.206	−	−	0.131	0.186	−	−	0.176	0.361	−	−
FGF21	0.198	0.047	0.247	0.053	0.276	0.009	0.543	<0.001	−0.087	0.617	−0.038	0.898
Waist	0.074	0.391	−0.118	0.363	0.082	0.395	0.071	0.650	0.035	0.857	−0.424	0.131
WHR	−0.030	0.730	−0.054	0.677	−0.004	0.967	0.074	0.637	−0.419	0.024	−0.463	0.096
SBP	0.006	0.947	−0.009	0.946	0.008	0.938	0.134	0.390	−0.174	0.366	−0.201	0.490
DBP	−0.018	0.836	−0.029	0.823	0.033	0.737	0.143	0.361	−0.114	0.558	−0.170	0.560
FBG	−0.031	0.724	−0.102	0.431	−0.140	0.151	−0.256	0.097	0.142	0.479	0.224	0.442
2hBG	0.026	0.768	−0.085	0.512	−0.108	0.269	−0.163	0.297	−0.039	0.846	−0.437	0.118
HbA1c	0.063	0.489	0.062	0.635	0.004	0.968	0.058	0.714	0.116	0.563	−0.029	0.921
TC	0.211	0.014	0.272	0.033	0.214	0.026	0.299	0.051	0.131	0.497	−0.005	0.987
TG	0.296	<0.001	0.213	0.097	0.325	0.001	0.231	0.137	0.002	0.994	−0.369	0.194
HDL-c	0.009	0.916	−0.087	0.502	−0.104	0.286	−0.152	0.330	0.345	0.067	0.276	0.340
LDL-c	0.059	0.492	0.201	0.117	0.091	0.348	0.193	0.215	0.067	0.729	0.121	0.679
APOA	0.200	0.028	0.211	0.099	0.199	0.053	0.285	0.064	0.118	0.564	0.089	0.762
APOB	0.199	0.028	0.277	0.029	0.241	0.019	0.350	0.021	−0.180	0.368	−0.090	0.759
APOE	0.252	0.011	0.226	0.078	0.154	0.177	0.184	0.238	0.021	0.923	0.041	0.890
ALT	0.436	<0.001	0.443	<0.001	0.378	<0.001	0.402	0.008	0.123	0.541	0.055	0.853
AST	0.487	<0.001	0.461	<0.001	0.373	<0.001	0.339	0.026	0.072	0.710	0.081	0.784
γ-GT	0.238	0.005	0.225	0.079	0.150	0.121	0.131	0.402	0.033	0.864	0.018	0.952
LDH	0.325	0.001	0.162	0.210	0.244	0.026	0.023	0.883	0.371	0.062	0.193	0.509

BMI: Body mass index;WHR: waist-to-hip ratio; SBP: systolic blood pressure; DBP: diasystolic blood pressure; FBG: fasting blood glucose; 2hBG: 2 h postload blood glucose. TC: total cholesterol; TG: triglyceride; HDL-c: high density lipoprotein cholesterol; LDL-c: low density lipoprotein cholesterol; APOA: apolipoprotein A; APOB: apolipoprotein B; APOE: apolipoprotein E; ALT: Alanine aminotransferase; AST: Aspartate aminotransferase; γ-GT: γ-glutamyl transpeptidase; LDH: lactate dehydrogenase.

*sex,age and BMI adjusted.

To compare the diagnostic value of FGF21 and other common clinical metabolic parameters in reflecting HFC, we conducted multivariate stepwise regression analysis between HFC and variables which are significant in univariate analysis and relevant to HFC, including: sex, FGF21, ALT, AST, γ-GT, LDH, TC, TG, APOA, APOB, APOE when HFC was in Q1–Q4, and age, FGF21, ALT, AST, LDH, TC, TG, APOB when HFC was in Q1–Q3. FGF21 has already been shown to be correlated with age in some studies [27], therefore, age also was adjusted in the multiple regression analysis when HFC was in Q1–Q4. We found that in all subjects, AST and sex(female) were independently associated with HFC (all *p*<0.05). However, when the subjects with the highest quartile of HFC were excluded from the analysis, FGF21 became the strongest factors independently associated with HFC (Table 3).

**Table 3:** Multiple stepwise regression analysis.

Independent variables	Standardized coefficient Beta	*p* value
Model 1 (HFC: Q1–Q4)		
AST	0.514	<0.001
sex	0.215	0.034
Model 2 (HFC: Q1–Q3)		
FGF21	0.409	<0.001
ALT	0.340	0.002

Model 1 HFC (Q1–Q4) was the dependent variable, independent variables were age and the variables which are significant in univariate analysis and relevant to HFC, including : sex(1 = M, 2 = F), FGF21, ALT, AST, γ-GT, LDH, TC, TG, APOA, APOB, APOE.

Model 2 HFC (Q1–Q3) was the dependent variable, independent variables were the variables which are significant in univariate analysis and relevant to HFC, including : age, FGF21, ALT, AST, LDH, TC, TG, APOB.

## Discussion

In the present study, we demonstrated the close association of serum FGF21 concentrations with intrahepatic fat content in 138 patients with abnormal glucose metabolism and with B ultrasound-diagnosed hepatic steatosis, whose hepatic fat content were distributed in a large range (2.47%–81.95%). To the best of our knowledge, this study is the first to show the quantitative correlation between serum FGF21 concentrations and hepatic fat content measured by ^1^H MRS in patients with impaired glucose metabolism. Interestingly, we found that in patients with mild or moderate hepatic steatosis (HFC was in Q1–Q3), FGF21 was the strongest factors independently associated with HFC among all metabolic parameters measured. However, when the hepatic fat content increased to the fourth quartile, serum FGF21 concentration no longer increased, but tended to decrease on the contrary.

A previous study has shown that in 17 patients with pathological liver triglycerides ranged from 10% to 40%, serum FGF21 concentration was highly positively correlated with hepatic fat content [20], similar to the results of our current study. Mounting evidences have suggested FGF21 as a protective metabolic regulator against a series of abnormalities in glucose and lipid metabolism. FGF21 is most abundantly expressed in the liver and can be directly induced by free fatty acids (FFAs), through PPARα, whose responsive elements had been found in the promoter regions of human FGF21 genes [28]. Liver is the main processing site of FFAs released from white adipose tissue (WAT). Therefore, hepatic cells are able to directly “sense” the alteration of circulating FFAs and regulate the concentration of FGF21 accordingly. A recent study has reported that circulating FGF21 level was closely related with the daily oscillation of free fatty acids [25], which also supported the FFAs-dependent activation of FGF21 in humans. Under the condition of obesity and insulin resistance, excessive influx of FFAs to the liver would induce FGF21 over-expression, and then elevated FGF21 could in turn decrease the level of serum FFAs through the inhibition of lipolysis in WAT [29]and inhibit the hepatic triglycerides generation and hepatic steatosis through promotion of fatty acid oxidation and ketogenesis [30]. Therefore, it is possible that the elevation of FGF21 is a hepatic protective response to the whole-body lipid metabolic burden influx to the liver, and the hepatic fat content directly reflect the excessive FFAs that enter the lipid synthesis pathway in the liver. Therefore, the serum FGF21 increases independently with the degree of hepatic steatosis to maintain a balance of hepatic lipid metabolism. In addition, since liver is the predominant organ for FGF21 production and action, it is possible that fat accumulated in the hepatic cell could also directly stimulate the secretion of FGF21 or cause an attenuated functional response to FGF21 (FGF21 resistance), thus leading to a compensatory FGF21 up-regulation.

Interestingly, when hepatic fat content increased to the fourth quartile, we found that the serum FGF21 concentration began to decrease on the contrary (Figure S1A). In line with our finding, a recent study reported that serum FGF21 levels were increased in individuals with NASH, but FGF21 level in NASH patients was much lower than that in NAFLD patients [21]. In the current study, in patients with hepatic fat content in the fourth quartile, the serum concentration of ALT, a well-established marker of hepatic injury, was also elevated (Table 1, Figure S1B), indicating the presence of hepatic injury in these patients. Therefore we speculated that the decrease of FGF21 in patients with severe hepatic steatosis might also be explained by the hepatic cell injury or death caused by lipoxicity and hepatic inflammation, so that the remaining hepatic cells were unable to produce as much FGF21 as needed. If our assumption turned out to be true, then a decrease of FGF21 level in NAFLD patient might indicate a decompensatory stage of the disease and might accompany with an acute deterioration of a series of metabolic disorders.

As we have shown that the FGF21 concentration in patients with mild or moderate hepatic steatosis was elevated in parallel with serum ALT level, but this balance would break in severe NAFLD patients, whose biochemical indexes will show an obviously elevated ALT concentration but only a slight unparallel elevation of FGF21 concentration probably due to the presence of hepatic injury. Therefore, it is possible that the insufficiency of FGF21 relative to elevation of ALT concentration might be a warning for hepatic cell injury clinically. Our study also found in patients with hepatic fat content no more than the fourth quartile, serum FGF21 was better than any metabolism-related parameters, including ALT, AST and TG, to reflect the hepatic fat content. Traditionally, ALT was most commonly used parameter to reflect hepatic impairment including NAFLD. However, our study indicated that FGF21 might be a better serum biomarker for NAFLD than ALT, though the clinical value of FGF21 as a NAFLD biomarker still need to be validated by further large-scale studies in the general population.

There are several limitations in this study. Firstly, since the study detected the quantitative correlation between FGF21 and hepatic fat content in a specific group of participants with abnormal glucose metabolism and B ultrasound-diagnosed hepatic steatosis, the average hepatic fat content in our subjects was much higher than the general population, so further studies are needed to determine the clinical value of FGF21 as a biomarker for NAFLD in the general population. Secondly, as a non-invasive imaging technique, ^1^H MRS can detect fatty infiltration of the liver, but unlike “gold standard” liver biopsy, it is limited in its ability to detect coexisting inflammation or fibrosis. However, in this article, we concerned more about the relationship of HFC with FGF21 and other metabolic parameters than the pathological changes of liver, and liver biopsy is an invasive examination which can not be accepted easily by patients, furthermore, it has been demonstrated that histology correlates well with ^1^H MRS in evaluating hepatic triglyceride content [31]. Several clinical trials [32], [33] on NAFLD have used ^1^H MRS as an outcome measurement. Therefore, ^1^H MRS may be a more appropriate reference standard than histology in accurately assessing fat content, especially in a relatively large sample study. Thirdly, we speculated the presence of hepatic injury in patients with severe hepatic steatosis according to the ALT concentration, a simple marker for hepatic injury, biopsy-proven data are needed to confirm the hepatic pathological features in severe NAFLD patients in our future works.

In summary, our study demonstrated that FGF21 was strongly correlated with the hepatic fat content in people with mild or moderate hepatic steatosis and could better reflect hepatic fat content than any known serum parameters. Furthermore, we found a decrease of FGF21 in patients with severe hepatic steatosis, which might indicate the presence of hepatic injury. These results support the role of FGF21 as a potential biomarker for NAFLD and further suggest an important role of FGF21 in regulating hepatic lipid metabolism in humans.

## Supporting Information

Figure S1 **Levels of serum FGF21 and ALT in patients with different HFC quartiles.** (A)serum FGF21 concentrations (pg/ml) (B) serum ALT levels (U/L). *: *p*<0.05, significant difference compared with group Q1; #: *p*<0.05, significant difference compared with group Q2. Compared with Q3, serum FGF21 of Q4 was decreased, *p* = 0.059.
(TIF)

Figure S2 **Association between serum FGF21 and HFC when (A) HFC was in the range of Q1–Q4; (B) HFC was in the range of Q1–Q3; (C) HFC was in the range of Q4.**
(TIF)

Supplement S1 **The detailed method of measurement of serum FGF21.**
(DOC)
