# Supplementary figures and images for: Circulating Fibroblast Growth Factor 21 Levels Are Closely Associated with Hepatic Fat Content: A Cross-Sectional Study
Source: PLoS One. 2011 Sep 16;6(9):e24895. doi: 10.1371/journal.pone.0024895 (PMC3174975; doi:10.1371/journal.pone.0024895)

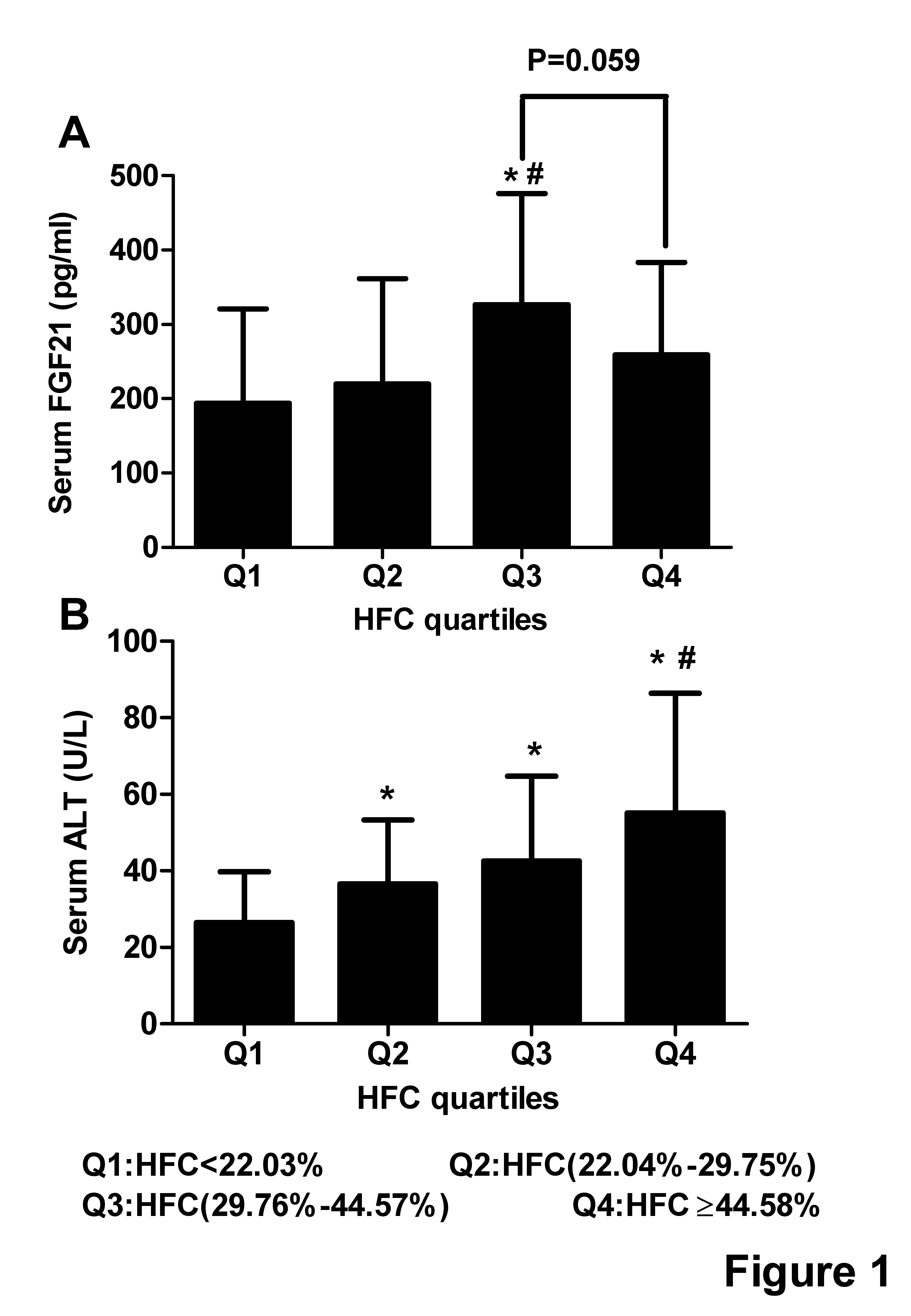

Supplement: Figure S1 — Levels of serum FGF21 and ALT in patients with different HFC quartiles. (A)serum FGF21 concentrations (pg/ml) (B) serum ALT levels (U/L). *: p<0.05, significant difference compared with group Q1; #: p<0.05, significant difference compared with group Q2. Compared with Q3, serum FGF21 of Q4 was decreased, p = 0.059. (TIF) [file pone.0024895.s001.tif]

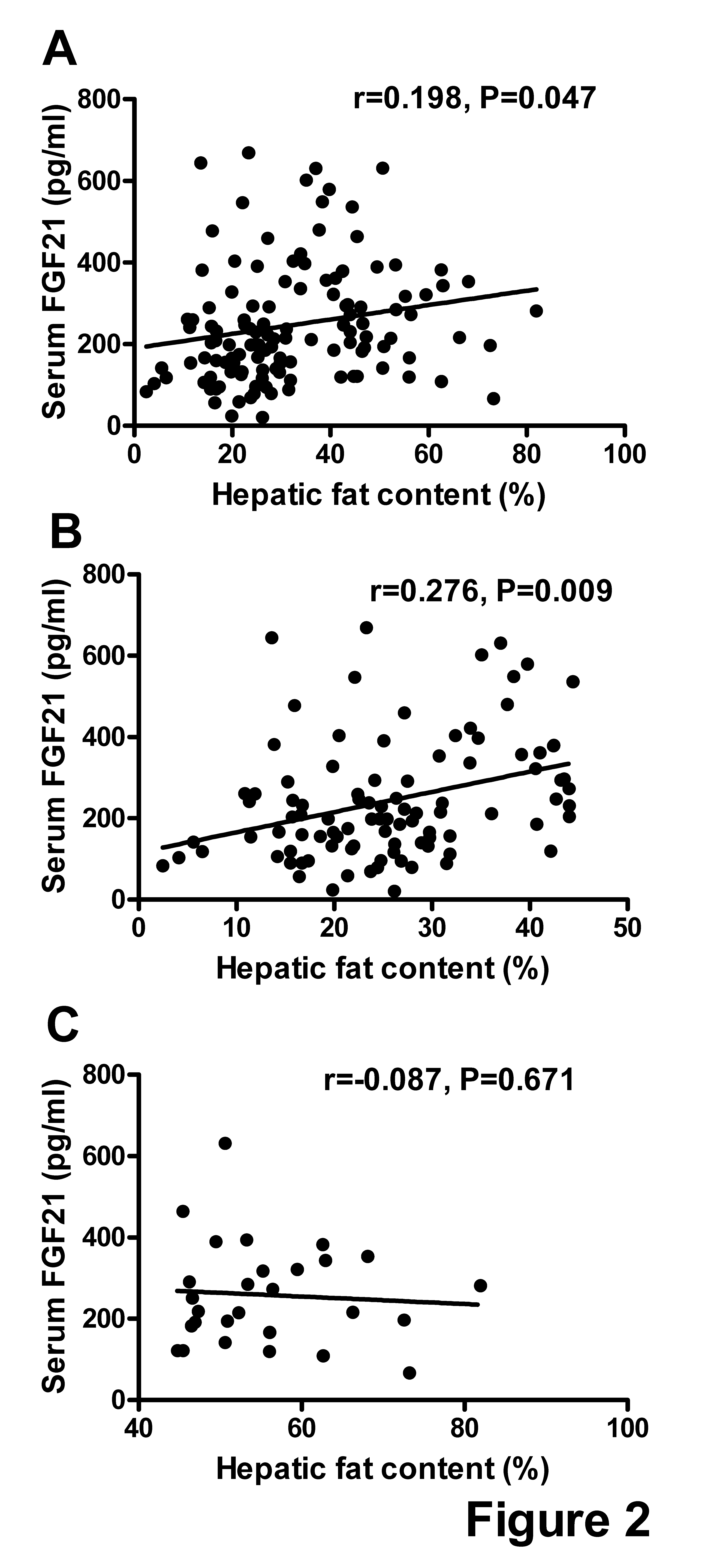

Supplement: Figure S2 — Association between serum FGF21 and HFC when (A) HFC was in the range of Q1–Q4; (B) HFC was in the range of Q1–Q3; (C) HFC was in the range of Q4. (TIF) [file pone.0024895.s002.tif]
