# Supplementary material for: Circulating Fibroblast Growth Factor 21 Levels Are Closely Associated with Hepatic Fat Content: A Cross-Sectional Study
Source: PLoS One. 2011 Sep 16;6(9):e24895. doi: 10.1371/journal.pone.0024895 (PMC3174975; doi:10.1371/journal.pone.0024895)
Supplement: Supplement S1 — The detailed method of measurement of serum FGF21. (DOC) [file pone.0024895.s003.doc]

**SUPPLEMENTAL MATERIALS AND METHODS**

**Expression of recombinant human FGF21 and generation of anti-human FGF21 antibodies**

The cDNA encoding human full length FGF21 (without signal peptide) was sub-cloned into pPROEX HTb vector (Invitrogen, Carlsbad, CA), and the recombinant FGF21 protein with His tagged at NH_2_-terminus was affinity-purified with Ni-NTA Sepharose (QIAGEN, Hong Kong). His-tag was then removed by digestion with TEV, and the recombinant human FGF21 was further purified with DEAE ion-exchange chromatography using FPLC (GE Healthcare, Pittsburgh, PA). The polyclonal antibodies against human FGF21 were generated by immunizing New Zealand female rabbits with the recombinant human FGF21 protein, and affinity-purified using recombinant FGF21 as a ligand.

The specificity of the anti-human FGF21 antibodies was confirmed by immunoprecipitation followed by tandem mass spectrometry as previously described (1). In brief, human serum was subjected to depletion of immunoglobulin and albumin with the ProteoExtract Albumin/IgG Removal Kit (Calbiochem, San Diego, CA) and the remaining supernatant was incubated with 500 μl of the Sepharose beads coupled with rabbit non-immune IgG to remove non-specific bindings. The clarified supernatant was then incubated with 500 μl of the Sepharose beads coupled with affinity-purified anti-human FGF21 antibodies at 4°C overnight. The beads were washed with PBS and the bound protein complexes eluted with 0.1 M glycine-HCl (pH 2.5). The affinity-purified FGF21 was digested by trypsin and analyzed by Orbitrap Velos mass spectrometer (Thermo, San Jose, CA). The identity of the serum FGF21 was confirmed by comparing the sequences of three peptides derived from tripson digestion with the published sequence of human FGF21 (NCBI accession number: NP_061986).

**Development of highly sensitive chemiluminescence immunoassay (CLIA) for human serum FGF21**

The high binding opaque polystyrene microplates (Corning Incorporated, NY) were coated with anti-human FGF21 antibodies at 4°C overnight in phosphate-buffered saline. After washing and blocking with 1% BSA, the recombinant human FGF21 standards or human serum samples were added, and incubated at room temperature for 1 hr. After washing three times, the biotinylated anti-human FGF21 antibodies were added and incubated for 1 hr at room temperature. The plates were then washed for three times, followed by incubation with Streptavidin-HRP (R&D System, Minneapolis, MN) for 30 min at room temperature. Finally, the chemiluiminescent substrate (Thermo Fisher Scientific, Rockford, IL) was added and incubated for 10 min, the luminescent signal (defined as relative light unit, RLU) was measured with a microplate luminometer. The concentration of human serum FGF21 was calculated based on the standard curve constructed by each concentration of the standards plotted with their corresponding RLUs. The intra-assay and inter-assay precision were determined by measuring two samples of known concentration on one plate ten times or in six separate assays respectively. The intra-assay and inter-assay coefficient of variation (CV) was 4.2- 5.6% and 5.8 - 7.3% respectively. The spiking recovery rate and linearity rate of the assay was 95% ~ 108% and 96% ~ 113% respectively.
